# Effects of Catalpol on Alzheimer's Disease and Its Mechanisms

**DOI:** 10.1155/2022/2794243

**Published:** 2022-06-30

**Authors:** Huize Chen, Chujun Deng, Zeyu Meng, Shengxi Meng

**Affiliations:** ^1^Department of Traditional Chinese Medicine, Shanghai Jiao Tong University Affiliated Sixth People's Hospital, Shanghai 200233, China; ^2^The Second Clinical Medical College, Heilongjiang University of Chinese Medicine, Harbin 150040, China

## Abstract

Alzheimer's disease (AD) is a degenerative disease of the central nervous system characterized by memory loss and cognitive dysfunction. With the increasing aging of the population, the incidence of AD and the number of patients are also increasing year by year, causing more and more heavy burdens to the family and society. Catalpol, an iridoid glycoside compound, is one of the main active components of *Rehmannia glutinosa*. At present, a large number of experimental studies in vivo and in vitro have confirmed that catalpol has antioxidant, anti-inflammatory, antiapoptotic, and other neuroprotective effects, and it plays a significant role in the prevention and treatment of AD, with very small side effects and high safety. Therefore, it may be an ideal drug for the treatment of AD. Based on this, the role and mechanism of catalpol in AD will be comprehensively reviewed in the following.

## 1. Introduction

**Figure 1:**
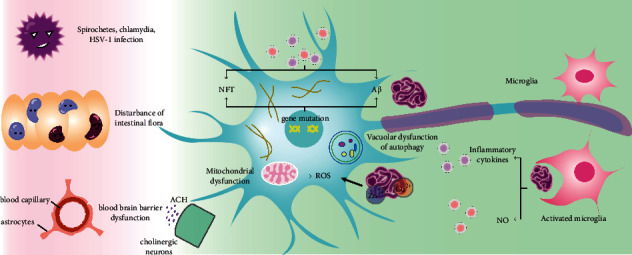
The pathogenic mechanisms and pathology of AD.

Alzheimer's disease (AD) is a neurodegenerative disease with insidious onset and progressive progression. Along with the increasing aging of the population in all countries, the prevalence of AD (see Table 1 for specific acronyms) is also increasing year by year. Currently, 6.2 million Americans aged 65 and older suffer from AD. It is expected to increase to 13.8 million by 2060 [1]. Memory impairment and cognitive decline are the main symptoms of early AD. The mechanism of Alzheimer's disease has not yet been fully clarified, but factors such as oxidative stress [2], mitochondrial dysfunction [3], and inflammation [4] play an important role in the occurrence and development of AD. Due to the complex pathogenesis (see Figure 1), current drug interventions for AD can improve symptoms to a certain extent but cannot prevent or delay the progression of AD [5].

**Figure 2:**
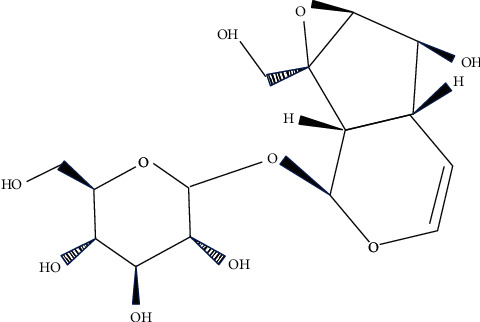
Chemical structure of catalpol.

**Figure 3:**
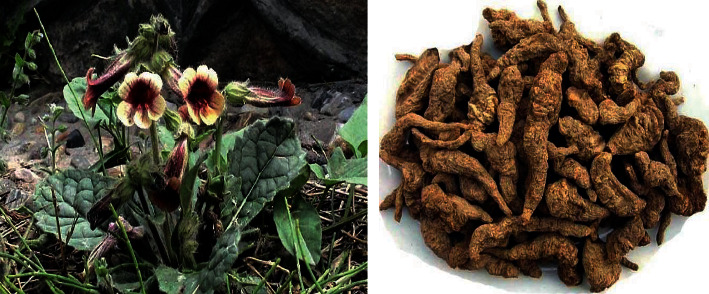
Plants and rhizome of *Rehmannia glutinosa*.

Traditional Chinese medicine (TCM) has the characteristics of multitarget action. Screening the active ingredients for AD from TCM is becoming a research hotspot. In recent years, A variety of Chinese herbal medicines have been proved to be effective in the treatment of AD, such as *Huperzia* [6], ginseng [7], and ginkgo [8], and some active ingredients (e.g., *β*-asarether [9], ligustrazine [10]) extracted from Chinese herbs medicines also have this effect. In addition, recent studies have shown that the traditional Chinese medicine compound Liuwei Dihuang Decoction can improve the learning and memory ability of mice with senile dementia, and catalpol, the main active ingredient in it, can inhibit the apoptosis of neural stem cells through the blood-brain barrier [11]. Catalpol (see Figure 2) is an iridoid glycoside compound extracted from the root of *Rehmannia glutinosa* (see Figure 3), which has been shown to have antioxidant, anti-inflammatory, and other neuroprotective effects, as well as certain protective effects against AD, PD, and neurological diseases such as hypoxic/ischemic injury [12]. In this paper, the effect and mechanism of catalpol on AD were reviewed.

## 2. Effect of Catalpol on Alzheimer's Disease

### 2.1. Anti-Inflammatory Effect

Under normal physiological conditions, inflammation is a defensive response against various injuries, which is composed of a series of molecules, cells, and their complex control network, helping to remove harmful substances and control the inflammatory process [13]. However, it is now considered to be a key factor leading to the progression of AD. In response to pathological substances (such as toxic proteins, dead neurons, etc.), the activation of glial cells can lead to the massive release of proinflammatory factors or other neurotoxic substances, leading to neuronal damage [14].

Yang C. et al. suggested that catalpol could significantly downregulate the levels of proinflammatory mediators nitric oxide and cytokines (interleukin and tumor necrosis factor *α*) in LPS-treated BV2 microglia. In addition, catalpol significantly reduced the levels of reactive oxygen species and malondialdehyde (MDA) in primary cortical neurons stimulated by hydrogen peroxide, increased the activity of superoxide dismutase (SOD) and glutathione (GSH), reversed cell apoptosis, and restored mitochondrial membrane potential (MMP) [15]. Transcriptional data also showed that catalpol significantly reduced the expression of inflammation-related genes, such as inducible nitric oxide synthase (iNOS), cyctoxase-2 (COX-2), and Toll-like receptor 4 (TLR4). Moreover, this study further revealed that catalpol's inhibitory effect on inflammation was achieved by inhibiting the activation of nuclear factor-*κ*B (NF-*κ*B). It is suggested that catalpol may inhibit the inflammatory response of astrocytes, and the inactivation of NF-*κ*B may be the main anti-inflammatory mechanism. Therefore, catalpol may be an effective drug for the treatment of inflammation-related neurodegenerative diseases [16]. Choi Yh et al. showed that catalpol significantly inhibited the secretion of proinflammatory mediators induced by LPS, including NO and prostaglandin E2. Meanwhile, catalpol also downregulates the expression of regulatory enzymes stimulated by LPS, such as iNOS and COX-2. Catalpol also inhibited the production and expression of LPS-induced proinflammatory cytokines, such as TNF-*α* and IL-1*β*. In addition, catalpol inhibits the NF-*κ*B signaling pathway by blocking the phosphorylation and degradation of *κ*B-*α* inhibitor and blocking the nuclear translocation of NF-*κ*B p65. Catalpol inhibited the LPS-induced expression of Toll-like receptor 4 (TLR4) and myeloid differentiation factor 88, which was associated with the inhibition of LPS binding to TLR4 on the cell surface. Catalpol significantly reduced the production of ROS induced by LPS. It is suggested that catalpol may inactivate the NF-*κ*B signal by antagonizing TLR4 and eliminating ROS, thereby inhibiting the inflammation of BV2 microglia mediated by lipopolysaccharide, and catalpol may play a potential role in the inhibition of the development and treatment of inflammatory diseases [17]. Glial cell-mediated inflammation plays an important role in the pathogenesis of AD. In vitro, besides having direct neurotoxic effects on neurons, A*β* also activates glial cells to produce a series of inflammatory factors, including TNF-*α*, ROS, NO, and iNOS, thus accelerating the progression of AD. Catalpol can protect neurons from damage caused by various toxic stimuli. Before 5 microM A*β*1-42, pretreatment with catalpol at 500 microM for 30 min not only attenuated the neurotoxicity of A*β*1-42-triggered neurons but also inhibited the activation of glial cells to a certain extent. Therefore, catalpol may be a useful anti-inflammatory agent for the treatment or prevention of inflammation-related neurodegenerative diseases, such as AD [18].

### 2.2. Antioxidative Stress

Dysfunction of neurons in specific regions caused by oxidative stress is an important part of the pathological process of AD. Oxidative DNA damage in neurons is closely related to cognitive deficits and occurs at the early stage of pathological changes in various neurodegenerative diseases [19]. The brain is more susceptible than other tissues to oxidative stress, which is involved in the development of AD by promoting A*β* deposition, Tau hyperphosphorylation, and neuronal damage. Therefore, improving the antioxidant effect may be beneficial for the treatment of AD [20, 21].

Tian YY et al. found that catalpol increased neuronal activity, significantly reduced the dose-dependent death of MPP + -induced dopaminergic neurons, and prevented the inhibition of complex I activity induced by MPP+ and the loss of MMP. In addition, catalpol reduces the content of lipid peroxides and improves the activities of GPX and SOD [22]. Rotenone significantly altered mitochondrial functions, such as complex I activity, decreased MMP, enhanced antioxidant status, glutathione depletion, and enzyme (GPX and SOD) disturbance, and increased lipid peroxidation. Catalpol increased the activities of complex I, SOD, and GPX in rotenone-treated mice and decreased the loss of lipid peroxidation and MMP. This indicates that catalpol has a certain protective effect on rotenone-induced toxic damage in mice [23].

Catalpol could significantly increase cell viability and reduce the formation of intracellular ROS. In addition, catalpol inhibited H_2_O_2_-induced oxidative stress by inhibiting the decrease of antioxidant enzyme activities such as GPX, GSH reductase, and GSH content during the GSH redox cycle. However, the promotion of catalase activity by catalpol was not obvious. The protective effect of catalpol on H_2_O_2_-damaged astrocytes may be related to maintaining the balance of glutathione metabolism and reducing the formation of ROS. Therefore, catalpol is a potential drug for the prevention or treatment of neurodegenerative diseases (such as AD) related to oxidative stress [24]. Catalpol can obviously improve cell morphology, enhance cell viability, and maintain the integrity of the cell membrane. In addition, catalpol could significantly inhibit the decrease of T-SOD and GSH-Px activities and the increase of MDA content in cells. Catalpol had a protective effect on oxidative damage of astrocytes induced by H_2_O_2_ [25].

Huang JZ et al. showed that catalpol could reduce the oxidative stress of the cerebral cortex by regulating the activity and concentration of ROS-related enzymes SOD, GPX, and glutathione catalase, but it could not reduce the oxidative stress by regulating MDA. Catalpol also reduces the levels of soluble A*β*40 and A*β*42 in the cerebral cortex, thereby inhibiting the formation of senile plaques [26]. After catalpol treatment, the learning and memory impairment of mice was alleviated by the Morris water maze test. This suggests that catalpol may be a potential drug for the treatment of AD [26].

Therefore, catalpol can ameliorate oxidative stress-induced neurodegenerative diseases and is a potential drug for the prevention or treatment of oxidative stress-related neurodegenerative diseases (such as AD) [22, 24, 26].

### 2.3. The Resistance to Apoptosis

Liang JH et al. treated cortical primary cultured neurons with A*β*1-42 to induce neuron injury and used it as an in vitro model of AD. Catalpol inhibits neuronal apoptosis by reducing intracellular ROS and Bax levels, MMP, and cytopigment C release, as well as regulating the activity and cleavage of Caspase-3 and Caspase-9 [27]. Chronic cerebral hypoperfusion is thought to be the cause of white matter lesions (WMLS), which can lead to cognitive impairment. Catalpol can significantly inhibit the inflammatory response of white matter and reduce the apoptosis of oligodendrocytes and myelin sheath injury [28].

### 2.4. Catalpol Promotes the Growth of Cortical Neuronal Axons

Synaptic loss is one of the common factors that lead to cognitive impairment; catalpol can significantly improve cognitive function in elderly male Sprague-Dawley rats. In primary rat cortical neurons with A*β* injury, catalpol can prolong the microtubule-associated protein 2 (MAP-2) positive neurons in length and increase the cerebral cortex and hippocampal synaptic proteins (dynamin 1, PSD-95) and synaptophysin expression. Catalpol may be a potential therapeutic agent for the treatment of cognitive disorders such as AD [29]. Intriguingly, 1-5 mg·mL^−1^ catalpol can significantly promote the growth of axons. With 2.5 mg mL^−1^, the axon growth was shortened when the dose was 5 mg mL^−1^. Catalpol had the strongest promoting effect on axon growth with 2.5 mg mL^−1^. Catalpol can promote the growth of axons of cortical neurons but not the survival of cortical neurons [30].

### 2.5. Antiaging Effect

Aging is an independent risk factor for the onset of AD. According to statistics, 10% of people over 65 years old suffer from AD, while 40% of people over 85 years old suffer from AD [31]. Some experimental results suggest that catalpol has an antiaging effect.

The study of Zhang XL et al. found that catalpol significantly improved the cognitive dysfunction of aging model mice, inhibited the loss of neurons in the hippocampus, and enhanced the exploratory behavior and passive avoidance response of aging mice [32, 33]. Catalpol can increase SOD, GSH-Px, and Na^+^-K^+^ATPase and Ca^2+^-Mg^2+^ATPase activity and reduces MAD level, indicating that catalpol plays an antiaging role by increasing the activity of endogenous antioxidant enzymes and reducing the generation of free radicals [32]. Catalpol also regulates the activities of endogenous antioxidants, glutathione, and lipid peroxides in the spleen and liver. The antiaging effect of catalpol is realized at least in part by promoting the activity of endogenous antioxidant enzymes and normalizing the energy disorder. Catalpol is worthy of a further preclinical study in the treatment of AD [34]. Catalpol can increase the activity of LDH, glutamine synthase (GS), and Na^+^-K^+^-ATPase and Ca^2+^-Mg^2+^-ATPase activity, reducing the activity of creatine kinase in the brain of D-galactose aging mice. Therefore, catalpol may be used as an antiaging agent against neurodegenerative diseases such as AD [34].

Liu *J* found that compared with young mice, the elderly rats significantly decreased such as synapses and GAP-43 levels, catalpol can improve the synapses of aged rats and GAP-43 levels and raised some important signaling proteins, catalpol can also improve the damaged neural plasticity and increase the aging rat survival neurons in the brain network of information storage, and catalpol improve age-related loss of neural plasticity by “normalizing” involved in signaling cascade of presynaptic protein [35].

These studies (Table 2) have confirmed the antiaging effects of catalpol.

## 3. Potential Mechanism of Catalpol in the Treatment of AD

### 3.1. Inhibition of Excessive Production of Reactive Oxygen Species

Catalpol can significantly increase the survival rate of primary astrocytes induced by H_2_O_2_ and decreased the intracellular ROS level [24]. Catalpol inhibits neuronal damage by increasing the activity of antioxidant enzymes such as GSH-Px and glutathione reductase during the glutathione redox cycle. An important mechanism of catalpol's protective effect may be related to the maintenance of the glutathione metabolic system and the inhibition of ROS production [37]. Catalpol significantly inhibited LDH release, MDA level, and GSH decrease. Catalpol reduces Hcy-stimulated ROS overproduction and inhibits transcription activation of NF-*κ*B [38].

### 3.2. Improve the Activity of Antioxidant Enzymes

Catalpol increases antioxidant activity in the brain, probably due to an increase in antioxidant enzyme activity. Catalpol can protect PC12 cells from apoptosis induced by hydrogen peroxide [39]. Several subsequent studies have shown similar antioxidant effects [40, 41]. For example, Zhang et al. found that catalpol improved memory impairment and upregulated antioxidant capacity in D-galactose-damaged mice. Catalpol increased the activity of SOD and GSH-Px in the cerebral cortex and hippocampus, decreased the level of MAD, and increased Na^+^-k ^+^ ATPase and Ca^2+^-Mg^2+^ATPase activity [32]. In addition to improving antioxidant enzymes, catalpol has the ability to improve energy metabolism [18].

Therefore, the important mechanism of the catalpol neuroprotective effect may be closely related to the promotion of antioxidant enzyme activity, the reduction of MAD level, and the prevention of mitochondrial dysfunction.

### 3.3. Inhibition of NO Formation

Metabolic disorders or excessive NO production in the brain are associated with the pathology of AD [42].

Free radical NO produced by activated glial cells is involved in a variety of physiological/pathological processes. NO and iNOS as inflammatory molecules can further increase IL-1*β* [36, 43]. Catalpol can inhibit the formation of NO and improve neurodegenerative diseases, including PD and AD [18]. A*β*1-42 induces excessive production of NO and iNOS by astrocytes, thereby reducing the survival of cortical neurons. Catalpol can effectively reduce A*β*1-42-induced neurotoxicity by reducing glial cell activation and inhibiting the production of inflammatory cytokines including TNF-*α*, ROS NO, and iNOS [18].

Rotenone can increase the production of NO and the level of iNOS, while catalpol can reduce the above two levels [44]. According to the analysis of ERK and JNK phosphorylation levels, catalpol significantly reduced NO levels and regulated the activation of ERK and JNK [44]. The results of morphology, immunocytochemistry, and flow cytometry showed that catalpol inhibited the apoptosis of primary neurons in the midbrain. ERK signaling pathway plays an important role in NO-mediated neuronal degeneration. Catalpol may inhibit neuronal apoptosis by regulating the increase of NO and iNOS in ERK-mediated neurodegenerative diseases [44]. Pretreatment of astrocytes with catalpol had negative effects on LPS + IFN-*γ* stimulation, NO and ROS formation, and iNOS activity. At the transcriptional level, catalpol also weakens the gene expression of some inflammatory cytokines, such as iNOS, COX-2, and TLR4 [16].

### 3.4. Inhibition of Mitochondrial Dysfunction

Mitochondrial dysfunction may occur early in oxidative stress response and is an important factor in the pathological mechanism of neurodegenerative diseases [45]. An in vivo model of LPS-induced inflammation suggests that LPS induces a loss of mitochondrial integrity (i.e., decreased MMP and increased osmotic transition pore opening). Given catalpol for 10 days prior to LPS injection, it can protect brain mitochondrial function by reducing MMP opening in the hippocampus and cerebral cortex [46]. Catalpol treatment can reduce the high permeability of BBB induced by fibrils A*β*1-42. In addition, catalpol inhibits apoptosis by A*β*1-42 induces through mitochondria-dependent and death receptor pathways, decreased levels of matrix metalloproteinases(MMPs), MMP‐2, MMP‐9, and AGEs receptors, and increased levels of tight junction proteins (ZO-1, occludin, and claudin-5), LDL receptor-associated protein 1, and P-glycoprotein. Catalpol also enhanced soluble A*β* elimination [43].

Catalpol can inhibit mitochondrial dysfunction in cell models and animal experiments. In MPTP-exposed midbrain neuron-astrocyte culture, catalpol reduces the accumulation of ROS and MMP, as well as intracellular CA^2+^, mitochondrial complex I activity, inhibits the opening of MPT pore, reverses MAO-B activity, and prevents astrocytes from MPP + -induced apoptosis to inhibit mitochondrial dysfunction [47]. Recent in vivo studies by Zhang et al. also showed that catalpol may play a role in the treatment of neurodegenerative diseases (such as AD) by preventing mitochondrial dysfunction in the cerebral cortex and hippocampus [34].

### 3.5. Inhibition of Cell Apoptosis

Apoptosis is considered to be an important factor in the pathogenesis of neurodegenerative diseases. Similarly, it is also accompanied by mitochondrial dysfunction, leading to excessive production of ROS, loss of MMP, and release of cytochrome C [48]. It maintains a balance between the expression of proapoptotic Bax and antiapoptotic Bcl-2, which plays an important role in protecting cells from apoptosis. Increased Bax expression can promote cell apoptosis, while upregulated Bcl-2 expression can inhibit cell apoptosis [49]. Caspases play a major role in the cascade of apoptosis through internal and external pathways, and both caspase-3 and caspase-9 are the executor of apoptosis in neurodegenerative diseases [50].

In the apoptosis of H_2_O_2_ induced PC12 cells, the expression of Bcl-2 was downregulated, and the expression of Bax was upregulated. Mitochondrial cytochrome C was released into the cytoplasm, Caspase-1 and Caspase-3 were activated, and PARP was cleaved. Catalpol can not only inhibit the downregulation of Bcl-2, the upregulation of Bax, and the release of mitochondrial cytochrome C into the cytoplasm but also inhibit the activation of Caspase-3 and the cleavage of PARP and finally inhibit the H_2_O_2_-induced apoptosis. Therefore, catalpol can inhibit H_2_O_2_-induced apoptosis of PC12 cells by regulating Bcl-2 family members and inhibiting cytochrome C release and caspase cascade activation [38]. The antiapoptotic mechanism of catalpol may be through the effective regulation of Bcl-2 and Bax expression. Catalpol inhibits the leakage of cytochrome c from mitochondria to the cytoplasm and weakens the activation of caspase-3 and the cleavage of PARP [38]. The primary cortical neurons treated with A*β*1-42 were used as AD cell models in vitro. After exposure to A*β*1-42 (5 microM) for 72 h, apoptosis occurred in the neurons, which were characterized by enhanced activity of caspases and ROS, increased Bax, loss of MMP, and release of cytopigment c. Pretreatment with 0.5 mM catalpol for 30 min followed by A*β*1-42 inhibited neuronal apoptosis by inhibiting ROS accumulation, Bax level, MMP, and cytopigment C release and regulating the activity and division of Caspase-3 and Caspase-9 to a certain extent. Therefore, catalpol plays a protective role in A*β*1-42-induced primary cortical neurons through the mitochondria-dependent caspase pathway [27].

Catalpol can protect cortical neurons from A*β*1-42-induced neurotoxicity [26]. This effect is partly related to the regulation of mitochondria-dependent caspases, the reduction of Bax expression and intracellular ROS accumulation, and the reduction of mitochondrial dysfunction.

Therefore, catalpol can inhibit nerve cell apoptosis and delay or prevent the cognitive decline caused by nerve cell apoptosis.

### 3.6. Regulating the NF-*κ*B Signaling Pathway

NF-*κ*B is an important transcription factor that is expressed in brain cells, including neurons, microglia, and astrocytes, and is involved in a variety of brain functions. In particular, glial NF-*κ*B has been identified as a key signaling molecule in neurodegenerative diseases (such as AD), brain injury, and viral infection [51, 52].

Catalpol may play a neuroprotective role by inhibiting the NF-kB signaling pathway to attenuate the microglia-mediated neuroinflammatory response. It blocks oxidative damage of cortical neurons by inhibiting the p53-mediated Bcl-2/Bax/caspase-3 apoptosis pathway and regulating the Keap1/Nrf2 pathway [15]. Catalpol significantly reduced the production of NO and ROS and the activity of iNOS. Moreover, catalpol can effectively reduce the expression of inflammatory-related genes, such as iNOS, COX-2, and TLR4. In addition, catalpol inhibited the inflammatory response by inhibiting the activation of NF-*κ*B. Catalpol can inhibit the inflammatory response of astrocytes, and the inactivation of NF-*κ*B may be the main determinant of its anti-inflammatory mechanism. Therefore, catalpol may be a very effective drug for the treatment of inflammation-related neurodegenerative diseases such as AD [16]. Catalpol inhibits NF-*κ*B signaling by reducing TLR4 and ROS levels, thereby inhibiting LPS-mediated inflammation of BV2 microglia [17].

### 3.7. Regulation of Neurotrophic Factors

The neurotrophic factor is a growth factor. They can prevent programmed cell death initiated by the associated neurons, thus facilitating neuronal survival [53]. Neurotrophic factors such as BDNF and GDNF have strong neuroprotective effects [54].

BDNF is an important factor regulating neural plasticity. It not only promotes neuronal survival and differentiation but also regulates synaptic plasticity and transmission in the central nervous system [55, 56]. BDNF may play a central role in the mechanism of synaptophysin affecting neuroplasticity [57]. The effect of catalpol on the cholinergic system was eliminated in vitro by blocking the action of BDNF by K252a or BDNF functional antibodies [58]. This suggests that catalpol may ameliorate the decline in memory function by improving partial cholinergic function, which is associated with BDNF activity. In neurodegenerative animals, morphological changes (e.g., reduced dendritic branching patterns, density of dendritic spines, and density of hippocampal fibers) are closely associated with decreased BDNF content in the brain [57, 59]. Therefore, the increased expression of BDNF after catalpol treatment is likely to play a key role in the improvement of learning and memory [60]. Catalpol also significantly increased the level of BDNF in the brain, thereby increasing the survival rate of new neurons by inhibiting apoptosis.

Catalpol improves memory by increasing BDNF expression and protects forebrain neurons from neurodegenerative diseases [58]. Compared with the elderly rats, the increase of PKC and BDNF in the hippocampus of the catalpol treated group was highly correlated with synaptophysin and GAP-43. These results indicated that catalpol could increase the presynaptic protein in the hippocampus of aged rats and upregulate the relevant signaling molecules. Therefore, catalpol may ameliorate age-related neuroplasticity loss by “normalizing” presynaptic proteins and their associated signaling pathways in elderly rats [35]. Catalpol can also significantly increase the level of BDNF in the brain, thereby increasing the survival rate of newborn neurons by inhibiting apoptosis [61].

### 3.8. Increasing the Density of Muscarinic Receptors in the Brain

In a mouse model of dementia, catalpol improves learning by increasing the density of muscarinic receptors in the brain [62]. Serum levels of ACh, ChAT, and BDNF in the catalpol group increased in a dose-dependent manner, while the level of AChE decreased in a *U*-shaped dose-corresponding curve. Catalpol significantly increased the levels of muscarinic AChR subtypes M1 and M2 in the hippocampus. This suggests that catalpol has neuroprotective and memory-enhancing effects, the mechanism of which may be related to the central cholinergic system [63]. Catalpol modulates cholinergic nervous system function through its effect on ChAT. Catalpol may be helpful in the treatment of AD but has no effect on *M* receptor affinity [64]. The activity of acetylcholinesterase (AChE) in the brain of aging mice was increased, the activity of ChAT positive neurons in the basal forebrain of aging mice was significantly decreased, and the expression of muscarinic acetylcholine receptor M1 (MAChR1) was decreased. It was also found that the levels of TNF-*α*, IL-1B, and advanced glycation end products (AGEs) increased in the brain tissues of aging mice. However, these biochemical indices were significantly reversed after two weeks of catalpol administration. It was suggested that catalpol had a protective effect on the brain of aging mice induced by *D*-galactose, which might be related to the protective effect of catalpol on the brain cholinergic and immune damage of mice. Therefore, catalpol is worthy of further use in preclinical studies of AD [65]. Catalpol can improve the structural abnormalities of the cerebral cortex and increase the expression of the M1 receptor in AD rats [66].

### 3.9. The Expression Levels of Bcl-2 and Bax Were Regulated

Catalpol stimulated the expression of Bcl-2 and inhibited the expression of Bax. Catalpol inhibited Ca^2+^ increased and downregulated CaMK phosphorylation in LPS-induced PC12 cells. CaMK-dependent ASK-1/JNK/*p*38 signaling cascades are blocked by catalpol and apoptosis is reduced [67]. Catalpol had an antiapoptotic effect on A*β*25-35-induced PC12 cells. Catalpol could increase the activity and decrease the apoptosis rate of PC12 cells damaged by A*β*25-35. At the same time, catalpol significantly inhibited A*β*25-35 induced, increased Bax expression, and decreased Bcl-2 expression [68]. Catalpol can significantly inhibit oligodendrocytes and myelin sheath damage and promote the recovery of cognitive decline. Catalpol also significantly increased the expression of Bcl-2 and phosphorylated cAMP response element-binding protein (p-CREB). In conclusion, catalpol can prevent hypoperfusion-induced white matter injury and cognitive impairment by upregulating Bcl-2 downstream through the P-CREB signaling pathway. It is suggested that catalpol may play a certain role in the treatment of cerebrovascular white matter injury [69].

### 3.10. Promoting PKC Expression

Catalpol significantly improved the cognitive function of aged rats and increase the expression of the synaptic protein (dynamin 1, PSD-95, synaptophysin) in the cerebral cortex and hippocampus. In addition, catalpol can prolong the length of MAP-2 positive neurons and reduce the inhibitory effect of A*β* on synaptophysin and synaptophysin in primary rat cortical neurons damaged by A*β*. Bisindolylmaleimide I, a PKC inhibitor, decreased the effect of catalpol on MAP-2-positive neurite growth and synaptic protein expression, suggesting that PKC may be involved in the prevention of A*β*-induced neurodegeneration by catalpol [29].

### 3.11. Protecting the Blood-Brain Barrier

The blood-brain barrier is crucial for maintaining the internal environment of the brain and its normal function. The destruction of the blood-brain barrier will accelerate the course of AD. In Alzheimer's disease (AD), excess A*β* deposition in the brain leads to cell damage and destruction of the blood-brain barrier (BBB). Liu CY study shows catalpol can inhibit A*β*1-42-induced brain microvascular endothelial cell apoptosis, increase levels of tight junction proteins to maintain the integrity of the blood-brain barrier, and can effectively regulate A*β*-related transporters on vascular endothelial cells to increase the clearance of soluble A*β* in the brain, which is a potential drug for the treatment of AD [70]. Feng S et al. also demonstrated that catalpol alleviated the increase of BBB permeability by inhibiting the decomposition of skeletal actin and connectin in BMECs, as well as the secretion of endothelin-1 and inflammatory cytokines [71].

### 3.12. Other Mechanisms

Catalpa promotes the expression of *α*-secretase (ADAM10) and its proteolytic products SAPP*α* and C83. In addition, the extracellular signal-associated kinase/cAMP response element-binding protein (ERK/CREB) signaling pathway is upregulated in catalpol treated SweAPP N2A cells. The effect of catalpol on the inhibiting A*β* generation might be closely involved with *α*-cleavage of APP processing [72].

**Figure 4:**
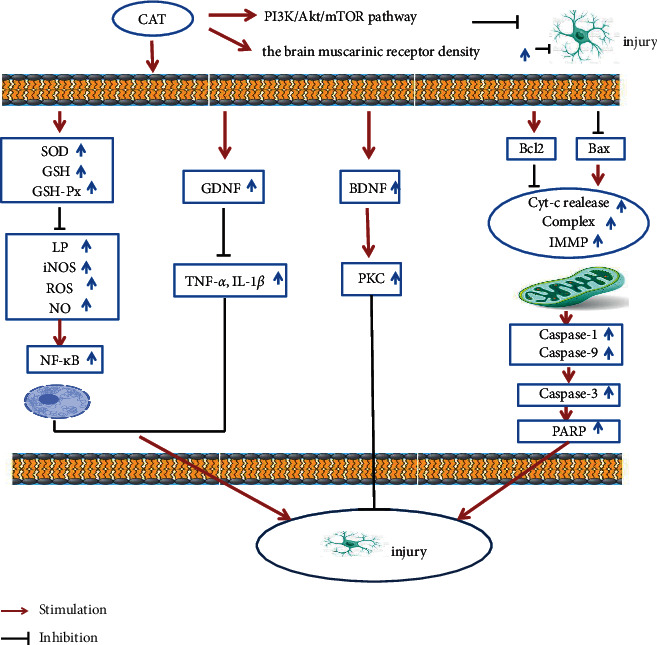
Catalpol mechanism diagram.

In summary, the mechanism of catalpol against AD is shown in Table 3 and Figure 4.

## 4. Conclusion

The pathogenesis of AD is very complex, involving cholinergic injury, immune inflammation, aging, and many other aspects. Disorder of intestinal flora may also lead to the progression of AD pathology and cognitive impairment [73, 74]. However, the drugs used for the treatment of AD are mainly single-target drugs, such as Donepezil hydrochloride and Memantine, which can improve or relieve the symptoms of AD patients to a certain extent, but patients are prone to some adverse reactions after medication, so it is not suitable for long-term use. TCM treatment of chronic diseases has the characteristics of integrity, multiapproach, and multitarget, with less toxic and side effects, and has a certain potential in the prevention and treatment of AD. TCM holds that insufficient kidney essence deficiency and the medullary sea are the underlying cause of AD; *Rehmannia glutinosa* with blood and nourishing Yin is beneficial to fill the effect of pulp; catalpol is the main active ingredient of *Rehmannia glutinosa*; on the animal and cell models, a large number of studies have shown that catalpol has significant nerve protection and anti-inflammatory and antiaging effect, and prompt catalpol has the potential to be the prevention and treatment of AD. Catalpol can not only protect neurons from injury but also promote the recovery of neuroendocrine disorders of the hypothalamic-pituitary-adrenal axis (HPA) in AD rats [75]. Catalpol has few side effects [76], and catalpol's small molecules have the ability to cross the blood-brain barrier [76]. With further research on the action and mechanism of catalpol, catalpol may become an ideal drug for the prevention and treatment of AD.

However, at present, there is very little clinical research data on the prevention and treatment of AD by catalpol, and the related studies are mainly focused on animal experiments and cell experiments, which need to be further strengthened and deepened in the future clinical studies. The system biology (such as metabolomics, etc.) and network pharmacology studies on catalpol prevention and treatment of AD are also insufficient, which will provide some evidence-based medical basis for explaining the mechanism, target, and network pharmacological target of catalpol.

However, it is important to note that pharmacokinetic studies have shown that catalpol has a short half-life. In in vivo studies, it lasts less than 1.5 hours [77, 78]. In order to prolong the half-life of catalpol and achieve a longer neuroprotective effect, the functional groups of catalpol will be modified in the future to screen and design more optimized catalpol analogs, so as to better prevent and control AD.

## Figures and Tables

**Table 1:** Abbreviation.

Acronym	Corresponding English
AD	Alzheimer's disease
AChE	Acetylcholinesterase
AGEs	Advanced glycation endproducts
BACE1	*β*-Secretase 1
BMECs	Brain microvascular endothelial cells
CAT	Catalpol
ChAT	Acetyltransferase
CK	Creatine kinase
COX-2	Cyclooxygenase-2
ERK	Extracellular signal-regulated kinase
EPO	Erythropoietin
GPx	Glutathione peroxidase
GSH	Glutathione
GSH-PX	Glutathione peroxidase
GS	Glutamine synthetase
H_2_O_2_	Hydrogen peroxide
IL	Interleukin
IDE	Insulin-degrad
iNOS	Inducible nitric oxide synthase
IRX3	Iroquois homeobox protein 3
LDH	Lactate dehydrogenase
LPS	Lipopolysaccharide
MDA	Malondialdehyde
MMP	Mitochondrial membrane potential
mAChR1	Muscarinic acetylcholine receptor M1
MAP-2	Microtubule-associated protein 2
MTT	3-(4,5-Dimethylthiazol-2-yl)-2,5-diphenyltetrazolium bromide
MS	Multiple sclerosis
NO	Nitric oxide
NF-*κ*B	Nuclear factor-*κ*B
NEP	Neprilysin
OGD	Oxygen-glucose deprivation
OLGs	Oligodendrocytes
p-CREB	Phosphorylated cAMP-responsive element-binding protein
PD	Parkinson's disease
PKC	Protein kinase C
PMA	Phorbol-12-myristate-13-acetate
PARP-1	Poly-ADP-ribose polymerase-1
ROS	Reactive oxygen species
SOD	Superoxide dismutase
TNF-*α*	Tumor necrosis factor *α*
TLR4	Toll-like receptor 4
TrkB	Tyrosine kinase receptor
VEGF	Vascular endothelial growth factor
WMLs	White matter lesions

**Table 2:** Effect of catalpol on anti-AD.

Category	Specific effects	References
The anti-inflammatory	(1) Downregulation of proinflammatory mediators NO and cytokines	[15]
	(2) Reduce the expression of genes associated with inflammation	[16]
	(3) Inhibit the production and expression of proinflammatory cytokines induced by LPS	[17]
	(4) Protects neuron cells from damage caused by various toxic stimuli	[18]

Antioxidant stress	(1) Prevent inhibition of complex I activity and loss of MMP induced by MPP	[22]
	(2) Increase the activities of complex I, SOD, and GPX, and reduce the loss of lipid peroxidation and MMP	[23]
	(3) Less H_2_O_2_-induced systemic oxidative stress	[24]
	(4) In view of the H_2_O_2_-induction of oxidative damage to astrocytes	[25]

Antiapoptotic	(1) Inhibition of nerve cell apoptosis	[27]
	(2) Reduce the apoptosis and myelin sheath injury of oligodendrocytes	[28]

Promote the growth of cortical neuronal axons	(1) Reduce the levels of soluble A*β*40 and A*β*42 in the cerebral cortex, thereby inhibiting the formation of age plaques	[26]
	(2) Enhance the clearance rate of soluble a*β*	[36]
	(3) Improve cognitive function and increase the expression of synaptic proteins	[29]
	(4) Promote the growth of cortical neuronal axons	[30]

Antiaging	(1) Antiaging effect	[32, 34]
	(2) Improve age-related loss of neuroplasticity	[35]

**Table 3:** Anti-AD mechanism of catalpol.

Category	Possible mechanisms (signaling pathway)	References
Inhibits excessive production of reactive oxygen species	(1) Increase cell survival and reduce intracellular ROS level	[24]

Improve the activity of antioxidant enzymes	(1) Improve the activities of SOD and GSH-Px	[32]
	(2) Improve the level of GSH and the activities of SOD and GSH-Px	[32]
	(3) Increase antioxidant enzyme activity	[18, 39]

Inhibition of NO production	(1) Negative effects on LPS + IFN-*γ* stimulation, NO and ROS formation, and iNOS activity	[16]
	(2) Inhibition of NO formation and improvement of neurodegenerative conditions	[18]
	(3) To prevent neuronal apoptosis by regulating the increase of NO and iNOS	[44]

Inhibits mitochondrial dysfunction	(1) Prevention of apoptosis induced by fiber A*β*1-42 through mitochondrial-dependent and death receptor pathways	[36]
	(2) Protecting brain mitochondrial function by decreasing MMP opening	[46]
	(3) Inhibition of mitochondrial dysfunction	[34, 47]
Inhibition of apoptosis	Attenuated mitochondria-dependent caspase cascade; inhibits the leakage of cytochrome C from mitochondria to the cytoplasm, and weakens the activation of caspase-3 and cleavage of polyADP ribose polymerase	[27, 39]

Regulation of NF-*κ*B pathway	(1) Inhibition of NF-*κ*B activation and LPS-induced acute inflammatory response	[43]
	(2) Inhibition of NF-*κ*B activation and protection of mitochondrial function	[46]
	(3) Reducing microglia-mediated neuroinflammatory response by inhibiting NF-*κ*B signaling pathway	[15, 16]

Regulation of neurotrophic factors	(1) *Hippocampus* BDNF was significantly increased, which was positively correlated with synaptophysin expression; “Normalize” presynaptic proteins and their associated signaling pathways	[35]
	(2) Enhance the expression of BDNF	[58, 60]

Increase the density of muscarinic receptors in the brain	(1) Protective effect on cholinergic	[49]
	(2) Improve learning ability and increase the density of muscarinic receptors in the brain	[62]
	(3) Regulating cholinergic nervous system function through the influence on ChAT	[64]

The expression levels of Bcl-2 and Bax were regulated	(1) Stimulate Bcl-2 expression and inhibit Bax expression	[67]
	(2) Inhibition of the increase of Bax expression and decrease of Bcl-2 expression induced by A*β*25-35	[68]
